# Routine outcomes and evaluation of an 8-week outpatient multidisciplinary rehabilitative therapy program for functional neurological disorder

**DOI:** 10.1007/s00415-023-12111-4

**Published:** 2023-12-13

**Authors:** Lisette Guy, Gabriella A. Caceres, Temeika Jackson, Sean Gorman, Jennifer Wilson, Yvonne Hsieh, Demelza Petty, Simon Harrison, Susannah Pick

**Affiliations:** 1 FiND Programme, Bethlem Royal Hospital, South London and Maudsley NHS Foundation Trust, London, UK; 2 Department of Psychological Medicine, Institute of Psychiatry, Psychology and Neuroscience, King’s College London, 16 De Crespigny Park, London, SE5 8AB UK

**Keywords:** Functional neurological disorder, Non-epileptic seizures, Functional seizures, Outpatient, Treatment, Intervention

## Abstract

**Objectives:**

We report routinely collected outcome data from an 8-week outpatient rehabilitative therapy program. The aims of the intervention were to (1) reduce symptom severity and (2) improve functional mobility in adults with functional neurological disorder (FND).

**Methods:**

The program delivered individual physiotherapy, cognitive behavioral therapy (CBT) and self-management sessions, group physiotherapy, and psychoeducation. Outcome measures included the Beck Anxiety Inventory (BAI), Beck Depression Inventory (BDI-II), Work and Social Adjustment Scale (WSAS), 10-Meter Walk Test (10MWT), Timed Up and Go (TUG), and Berg Balance Scale (BBS). Data were analyzed retrospectively in accordance with routine service evaluation. Wilcoxon signed-rank tests assessed changes in outcomes between weeks 1 and 8 for all patients completing treatment (*n* = 45). For patients who attended the 3-month follow-up (*n* = 31), Friedman’s ANOVA assessed overall change in outcomes over time. Post hoc Wilcoxon signed-rank tests compared pairs of time-points (Weeks 1, 8, and 3-month follow-up).

**Results:**

Analyses of patients completing the program revealed significant improvements in scores between week 1 and week 8. Excluding the BBS, there were statistically significant improvements in all outcomes between weeks 1 and 8 and between weeks 1 and 3-month follow-up.

**Discussion:**

This outpatient therapy program provided effective treatment for FND. Patients reported reduced anxiety, depression, and functional impairment, as well as improved performance on most physiotherapy measures.

**Supplementary Information:**

The online version contains supplementary material available at 10.1007/s00415-023-12111-4.

## Introduction

Functional neurological disorder (FND) refers to genuinely experienced neurological symptoms that cause significant distress or disruption, without evidence of an underlying neurological, psychiatric or other medical explanation [1, 2]. FND has an estimated incidence of 4–12 per 100,000 population per year [3], with women representing 60–75% of the FND population [4]. Functional symptoms are amongst the most common presentations in neurology outpatient settings, at approximately 15–16% of patients seen [5, 6]. FND can include multiple symptom domains, the core of which relate to motor function, weakness or paralysis, sensory disturbances, and episodes of altered awareness. Broader spectrum physical symptoms are common, as are accompanying psychological comorbidities such as depression, anxiety, post-traumatic stress disorder, and dissociative symptoms [7].

Individuals with FND tend to display high levels of disability and significant impairment in daily functioning [8]. FND can also be challenging to diagnose, as the symptoms can mimic those of other neurological disorders such as epilepsy, stroke, multiple sclerosis, or Parkinson’s disease; multiple investigations are often required. Individuals with FND, therefore, may undergo various medical procedures and wait a significant time prior to diagnosis, with a reported diagnostic delay in functional seizures (FS) of around seven years [9, 10]. The economic costs of healthcare resource use in FND are significant and comparable to other major neurological disorders [11]. However, at present, widely agreed etiological explanations remain elusive, and there is no clear consensus on the most effective treatment approach [12].

Contemporary explanatory models provide insight into attentional dysregulation, which is considered a major feature in FND [13]. The ‘free-energy principle’ suggests sensations (exteroceptive, interoceptive, proprioceptive) are constructed to minimize ‘sensory surprise’ [14]. According to this model, functional symptoms may arise from abnormal priors given excessive precision by attention, thus disrupting an automatic process [15]. Another model of FND incorporates both psychosocial and neurobiological approaches, with emphasis on the possible bridging role of emotional processing, autonomic arousal and limbic/paralimbic hyperactivation [16]. Together, contemporary models highlight the potential heterogeneity of etiological factors and pathophysiological mechanisms in FND and suggest that psychosocial and biological vulnerabilities may predispose an individual to FND via adaptations in attentional, somatosensory, and emotional processing [17].

The complex etiology and mechanisms of FND imply that an individual with the diagnosis may require input from a range of healthcare services. There are calls for improved integration between psychology, psychiatry, and neurology services for FND diagnosis and management [13, 18–20]. Interventions that include both physical and psychological aspects may be particularly beneficial [21], with reported improvements in FND symptoms following both physiotherapy and psychotherapy [22–26], as well as multidisciplinary team (MDT) approaches [12, 27, 28]. Provision of cognitive behavioral therapy in addition to standard medical care for functional seizures may also improve a variety of comorbid symptoms [29], which could be influential in terms of perceived quality of life [30].

Outcomes from MDT programs have been favorable, with reported improvements in Clinical Global Impression (CGI) scores and psychological symptoms [31, 32]. Both quality of life and ability to walk significantly improved in those with functional gait disorder following a 3-week MDT intervention [33]. A follow-up study found that 58% of patients with motor FND reported improvements in symptoms and overall function after attending a specialist MDT inpatient unit 2 years prior [34] and suggests that MDT approaches may be effective in producing long-term benefits. Findings from a day program demonstrated improvements in an array of outcomes [35], supporting the notion that outpatient interdisciplinary working may be beneficial [31, 32].

Given this background, the Functional integrated Neurological Disorders (FiND) program was developed. The program aimed to address a broad range of factors relevant to FND, while tailoring the service to meet individual needs and address symptom heterogeneity. Primary aims of the intervention were to (1) reduce FND symptom severity and (2) improve patient’s functional mobility. The FiND program is particularly suited to individuals whose symptoms do not warrant inpatient admission, thus supporting inpatient services.

In this retrospective service evaluation, we aimed to assess the effectiveness of the FiND program, in terms of the potential beneficial effects of the intervention in this real-world clinical setting [36]. We assessed this by examining routinely collected outcome data in the domains of sensorimotor functioning (mobility, balance), psychological distress (anxiety, depression), and functional impairment (work, social). These outcomes were compared between the start and end of the program, and at a 3-month follow-up where possible. We also sought to review limitations of the current program with the aim of implementing future improvements in service delivery.

## Methods

### Participants

Routine outcome measures were collected in accordance with standard service delivery. Patients with FND (*n* = 50) attended the 8-week outpatient program between late 2018 and early 2020. Data were collected throughout program attendance and subsequent follow-up sessions. Only data from patients who completed the program were included in the analyses (*n* = 45).

The reported analyses were conducted retrospectively to evaluate the effectiveness of the routine delivery of the FiND service. Therefore, according to the UK Health Research Authority, this project did not require approval by a NHS research ethics committee (https://www.hra.nhs.uk/approvals-amendments/). Patients were informed about outcome data collection and service evaluations on the first day of the program.

### Referrals

Referrals were accepted nationally from healthcare professionals such as consultants, general practitioners (GP) and mental health teams. Referrals were screened by the MDT prior to assessment. All patients were required to have a confirmed and accepted diagnosis of FND, made by a consultant neurologist or neuropsychiatrist according to DSM-5 criteria [1], with needs for both psychological and physiotherapy intervention, including those with functional motor, sensory and seizure symptoms. Patients were required to live within a 40-mile radius of Bethlem Royal Hospital to allow for travel time. Those who did not meet this requirement received a telephone call to discuss the option to arrange nearby accommodation for their designated days of attendance during the program, provided this was feasible. Suitability assessments were conducted in-person by the CBT therapist and the physiotherapist following the criteria outlined in Table 1. For patients who did not attend the program, the clinical pathway they were directed to was determined on an individualised, case-by-case basis.

**Table 1:** Suitability assessment criteria for program attendance

(1) Patient is accepting of FND^a^ diagnosis
(2) Patient is able to travel to and from the program for the duration of attendance
(3) Patient agrees to strive for 100% attendance—even if symptoms make this difficult
(4) Patient has no outstanding diagnostic or medical investigations
(5) Patient consents to reduce reliance on aides and adaptations
(6) Patient consents to a medication review
(7) Patient agrees that emergency services will not be called for identified functional symptoms
(8) Patient is able to transfer independently to and from a taxi
(9) Patient is able to attend to all self-care requirements
(10) Patient is not actively psychotic
(11) Patient is not actively suicidal

^a^*FND* Functional Neurological Disorder

### Program structure and delivery

Each cohort included three patients who attended the program for two days per week over eight weeks. The program was led by the FiND MDT, based at Bethlem Royal Hospital, London, UK. The outpatient unit provided a fully equipped physiotherapy gym and a private one-to-one therapy room for confidential CBT sessions. Each patient received individual hour-long sessions of CBT (× 16), physiotherapy (× 16) and self-management (× 16), along with group-based (*n* = 3) psycho-educational sessions, physiotherapy workshops, and mindfulness relaxation exercises. The program also included an ‘alumni’ session in week two and a ‘friends and family’ session in week five. The alumni session invited previous program patients to speak with current patients about their experiences of the program. For the friends and family session, patients could invite their loved ones to attend an educational session about the program. Once a cohort was concluded, patients were invited to return for follow-up sessions at one and three months.

#### Cognitive behavioral therapy

The CBT sessions were structured on the basis of formulations developed between the therapist and patient to identify patterns of behavior which potentially had an impact on recovery and symptom management. CBT models within a biopsychosocial framework (Five Areas Approach) [37] were used to challenge unhelpful beliefs and thinking styles, with a focus on responses to stressful situations. These sessions considered individual predisposing, precipitating and perpetuating factors. Patients were encouraged to monitor their activities, thoughts, emotions and behaviors through diaries, which helped identify and confront areas for change. Prevalent themes for sessions included overcoming anxiety responses, challenging avoidant behaviors, behavioral experiments and gradual exposure, education regarding therapeutic techniques such as grounding and attention training, and goal-directed activities.

#### Self-management

The focus of these sessions was to implement self-management techniques, such as gradually increasing activity levels, identifying and overcoming obstacles of inactivity, identifying potential symptom triggers, and effective time management with regard to ‘boom and bust’ activity patterns. Using diaries enabled the identification of a reference baseline of activities and monitoring of planned and scheduled activities to promote routines and engagement in daily activities for positive change. The themes of these sessions included: behavioral activation and gradually increasing activity, managing ‘boom-and-bust’ patterns, goal setting, strategies to overcome worry, managing anxiety, sleep hygiene and fatigue management, communication styles, volunteering, and relapse prevention planning.

#### Physiotherapy

Physiotherapy for FND is guided by principles emphasizing task-based interventions aiming to retrain movement and normalize sensory experience. Working collaboratively with each patient, goals to improve mobility and function were established. Sessions focused on retraining functional movements whilst using strategies to divert attention away from symptoms and toward task-based or cognitive activities. Patients were also given psycho-educational sessions relating to pain and the benefits of exercise. Graded exercise and pacing were the primary strategies implemented to manage fatigue and pain.

### Outcome measures

The variable nature of FND symptoms over time may make it difficult to assess the efficacy and effectiveness of treatment approaches [22]. However, expert recommendation suggests use of pre-existing, valid, and reliable measures across several key outcome domains, including core FND symptoms, other physical and psychological symptoms, life impact (e.g., disability, quality of life, global functioning), and health economics [7, 38]. Given the current lack of a well-validated and endorsed FND-specific outcome measure [38], the following combination of existing patient- and clinician-rated outcome measures were collected for each patient at the start (week 1) and end of the program (week 8), and at 3-month follow-up.

#### Patient-rated measures

The Beck Anxiety Inventory (BAI) [39] was used to quantify anxiety. The BAI is a psychometrically validated 21-item measure of anxiety symptoms by severity. Scores are classified as minimal anxiety (0–7), mild anxiety (8–15), moderate anxiety (16–25) and severe anxiety (26–63).

The Beck Depression Inventory (BDI-II) [40] was used as a measure of clinical depression. The BDI-II is a psychometrically validated 21-item scale to measure symptoms of clinical depression. Total score classifications indicate minimal depression (0–13), mild depression (14–19), moderate depression (20–28), and severe depression (29–63).

The Work and Social Adjustment Scale (WSAS) monitored self-reported functional impairment. The WSAS demonstrates sufficient psychometric properties [41, 42] and comprises five statements, which are rated on a scale of 0–8 indicating ‘no impairment at all’ to ‘very severe impairment’. Total score classifications indicate subclinical population (0–9), significant functional impairment (10–20), and functional impairment (> 20).

#### Clinician-rated measures

As all patients attending the FiND service had physiotherapy needs, physiotherapy outcome measures included the following clinician-rated tools: the 10-Meter Walk Test (10MWT) [43], Timed Up and Go (TUG) [44], and the Berg Balance Scale (BBS) [45]. As a descriptive measure, we documented the utilization of mobility aids, which could include the use of walking sticks, crutches, wheelchairs, and/or mobility scooters across one or more distinct environments.

The 10MWT and the TUG measured the time it took to mobilize and walk a certain distance. These tasks were completed either aided or unaided depending on the level of gait and balance function at the time of testing. Both the 10MWT and the TUG are considered to be psychometrically valid and suitable for use in clinical environments [46, 47].

The Berg Balance Scale (BBS) is a 14-item scale used to identify the ability to balance during a series of tasks and is recommended for use in adult populations [48]. Each item is rated from 0 to 4, with a maximum score of 56 which indicates functional balance, with scores less than 45 indicating a high risk of falls.

### Data analysis

Categorical outcomes were examined with chi-squared tests. We conducted Wilcoxon Signed-rank tests to compare scores between week 1 and week 8 for all patients who completed the program (*n* = 45).

To test whether we had an inclusion bias, we compared the sample of patients for whom we had 3-month follow-up data with the sample of patients whom did not attend the follow-up, using Mann–Whitney *U* tests. We then compared outcomes between weeks 1, 8 and 3-month follow-up using Friedman’s ANOVA (Kendall’s W effect size), with post hoc Wilcoxon Signed-rank tests for patients who attended the follow-up. Bonferroni corrections were applied to post hoc tests to allow for multiple comparisons and effect sizes were calculated from standardised test scores to provide values for the rank-biserial coefficient *r*. Effect sizes were interpreted in the same frame of reference as proposed by Cohen [49], with 0.1, 0.3, and 0.5, signifying small, moderate, and large effect sizes, respectively. Data collected from patients who did not complete the program were excluded given that end of program outcomes were not available for these patients.

## Results

### Sample

**Fig. 1:**
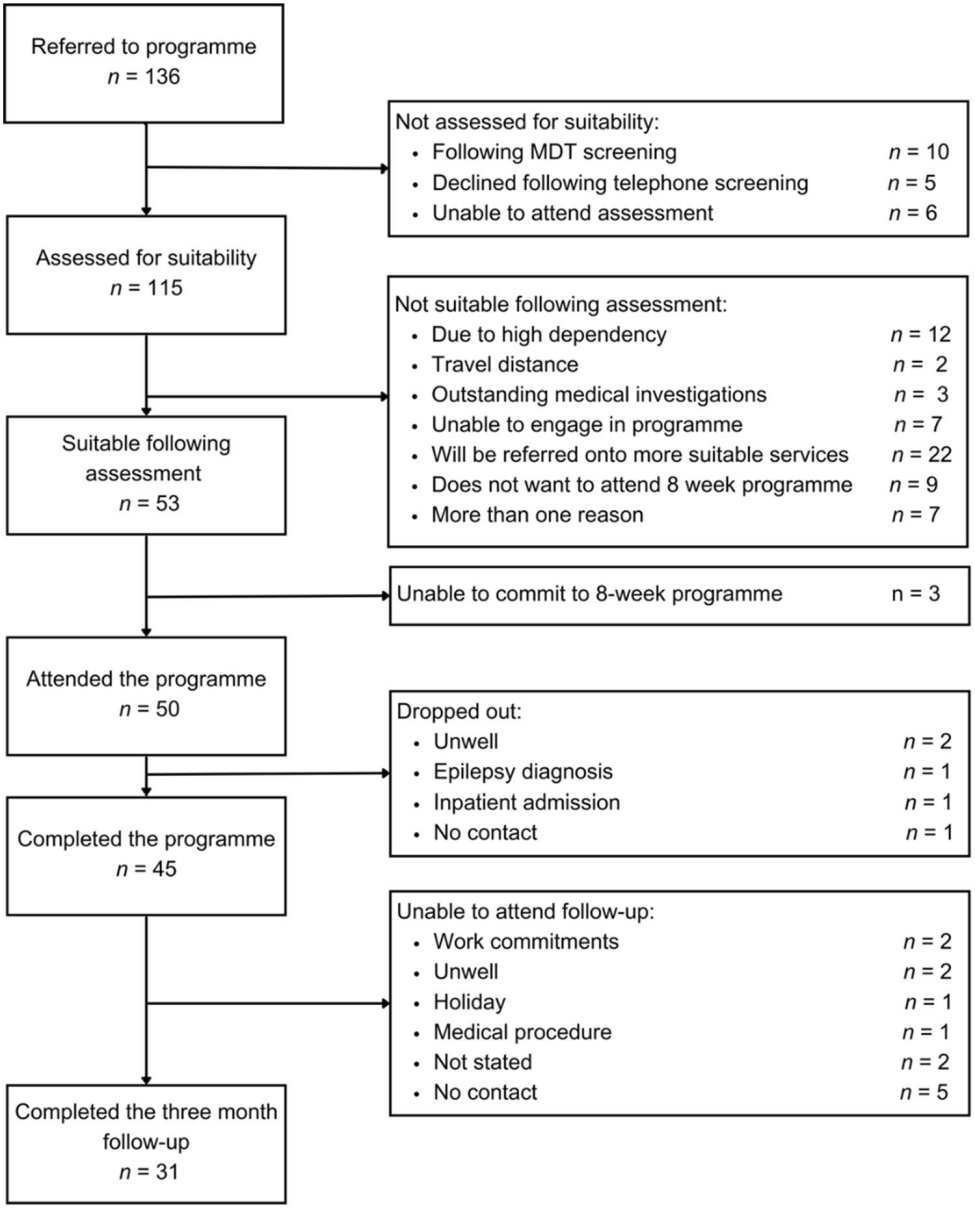
Patient referrals, assessments, and attrition (*n*)

Following suitability assessment, patients (*n* = 53) were invited to attend the program. As shown in Fig. 1, three patients were not able to attend, five patients were not able to complete the program, and a further 14 were unable to attend at 3-month follow-up. Data were included in analyses for all patients who completed the program (*n* = 45). The program did not have a physiotherapist in post for a 3-month period from the end of November 2019. Although all patients received physiotherapy sessions during the program, physiotherapy outcome measures were not completed for some individuals (*n* = 10).

### Baseline characteristics

Information regarding patient demographics and predominant symptoms for patients who completed the program is shown in Table 2.

**Table 2:** Predominant symptoms of all included patients at baseline (*n* = 45)

*Age*		
Mean age at onset (range)	39.95 years (16–61)	
Mean age at attendance (range)	44.69 years (24–65)	
Symptom duration (range)	4.74 years (0.3–21)	

	*N*	Percentage of sample (%)
*Sex*		
Male	8	16
Female	42	84
*Predominant symptoms*		
Weakness^a^	32	64
Left lower limb weakness	26	52
Right lower limb weakness	14	28
Left upper limb weakness	22	44
Right upper limb weakness	10	20
Functional seizures	18	36
Other motor symptoms^b^	29	58
Other sensory symptoms^c^	22	44
*Additional symptoms*		
Fatigue	31	62
Persistent pain	32	64
Headaches	13	26
Dizziness	7	14
Dissociative episodes	7	14
Cognitive difficulties	18	36
Speech difficulties	9	18
Falls	13	26
Tremors	11	22
Balance difficulties	12	24

^a^Includes bilateral upper and lower limb weakness

^b^Includes involuntary movements, dystonia, tremors, and balance problems

^c^Includes visual symptoms, dizziness, and tingling

At baseline, the symptoms most commonly reported for all patients (*n* = 50) were motor symptoms such as involuntary movements, dystonia, tremor and balance difficulties (64%), and weakness (70%), particularly left lower limb weakness (58%). Pain (72%), fatigue (64%), functional seizures (38%), and sensory symptoms were also common (e.g., visual symptoms, dizziness, numbness and tingling sensations, 48%). Thirty-nine patients (78%) had one or more psychiatric commorbidity at baseline (e.g., depression, obsessive compulsive disorder, post-traumatic stress disorder, personality disorder). None of the patients had a diagnosis of psychosis. Forty-one (82%) patients were taking either psychotropic or pain medications, with 37 (74%) patients taking one or more psychotropic medications (i.e., antidepressant, antiepileptic), and 29 (58%) patients taking one or more pain medications (including opioid agonists). Additionally, 36 (72%) patients were taking other medications (i.e., blood pressure, diabetes, and/or anti-inflammatoy medications).

At baseline (week 1), the majority of patients scored in ‘severe’ categories on the BAI (56%), BDI-II (58%), and WSAS (85%), suggesting that upon initial attendance to the program, patients generally experienced severe anxiety, severe symptoms of depression, and demonstrated severe functional impairment.

### Outcomes

Patients who completed the 8-week program demonstrated significant improvements in all outcome measure scores compared to baseline scores, as indicated by the Wilcoxon signed-rank test (Table 3). Descriptive statistics indicated that, of the patients who completed the 8-week program, 39% of patients could mobilize unaided prior to program attendance, with 67% able to mobilize unaided at week 8.

**Table 3:** Group median (interquartile range) changes in outcome measures scores between week 1 and week 8

	*N*	Week 1	Week 8	Statistic^*1*^ (*W*, *p* value)	*Z*	Effect size *r*
BAI	45	27 (18)	17 (15)	116.5, *p* < 0.001	− 4.31	0.64
BDI-II	45	31 (18)	14 (19)	81.5, *p* < 0.001	− 4.83	0.72
WSAS	45	27 (9)	21 (15)	102.0, *p* < 0.001	− 4.59	0.68
10MWT (m/s)	35	0.73 (0.58)	0.92 (0.54)	31.0, *p* < 0.001	− 4.36	0.74
TUG (s)	35	15 (15.37)	9.98 (8.43)	60.0, *p* < 0.001	− 4.06	0.69
BBS	35	48 (29)	54 (12.5)	64.0, *p* = 0.005	− 2.83	0.48

*BAI* Beck Anxiety Inventory,*BDI-ii* Beck Depression Inventory-Version ii, *WSAS* Work and Social Adjustment Scale, *10MWT* 10-Meter Walk Test, *TUG* Timed Up and Go,* BBS* Berg Balance Scale

^1^Denotes statistical significance following Wilcoxon signed-rank tests

Thirty-one patients attended the 3-month follow-up. The percentage of patients in the severe category on the patient-rated outcomes had reduced (Table 4). There were significant reductions in the proportion of patients scoring in the severe categories across time-points for the BAI (*χ*^*2*^ (2) = 8.00, p < 0.05), BDI-II (*χ*^*2*^ (2) = 7.85, p < 0.05), and WSAS (*χ*^*2*^ (2) = 9.30, p < 0.05).

**Table 4:** Self-report measure score category changes across time-points for patients who completed the follow-up (*n* = 31)

	Week 1	Week 8	3-Month follow-up
	*N* (%)	*N* (%)	*N* (%)
*BAI*			
Minimal Anxiety (0–7)	0 (0)	5 (16)	6 (19)
Mild Anxiety (8–15)	7 (23)	6 (19)	5 (16)
Moderate Anxiety (16–25)	3 (10)	10 (32)	6 (19)
Severe Anxiety (26–63)	21 (68)	10 (32)	14 (45)
*BDI-II*			
Minimal Depression (0–13)	1 (3)	14 (45)	10 (32)
Mild Depression (14–19)	2 (6)	2 (6)	2 (6)
Moderate Depression (20–28)	7 (23)	4 (13)	7 (23)
Severe Depression (29–63)	21 (68)	11 (35)	12 (39)
*WSAS*			
Subclinical Population (0–9)	1 (3)	7 (23)	6 (19)
Significant Functional Impairment (10–20)	2 (6)	6 (19)	6 (19)
Severe Functional Impairment (> 20)	28 (90)	18 (58)	19 (61)

*BAI* Beck Anxiety Inventory, *BDI-II* Beck Depression Inventory-Version ii, *WSAS* Work and Social Adjustment Scale

At week 8, patients who did not complete the 3-month follow-up had lower scores in the BAI and 10MWT than patients who attended the follow-up (Table 5). There were no significant differences in scores on the BDI, WSAS, TUG, or BBS at week 8 between patients who did and did not attend the 3-month follow-up (Table 5).

**Table 5:** Week 8 differences between patients who completed the follow-up and patients who did not complete the follow-up

	Completed FU^*1*^		Did not complete FU^*1*^		Statistics^*2*^	*Z*	Effect size
	*N*	Median (IQR)	*N*	Median (IQR)	(*U*, *p* value)		*r*
BAI	31	19 (20.5)	14	10 (8.5)	117.5, *p* < 0.01	− 2.44	0.36
BDI-II	31	19 (26.5)	14	11.5 (7.5)	158, *p* = 0.153	− 1.45	0.22
WSAS	31	22 (16.5)	14	14.5 (10.75)	168.5, *p* = 0.241	− 1.19	0.18
10MWT (m/s)	21	1.13 (0.45)	13	0.73 (0.37)	46, *p* < 0.01	− 3.21	0.55
TUG (s)	21	8.21 (6.15)	13	14.5 (9.58)	90, *p* = 0.105	− 1.65	0.28
BBS	21	56 (11)	13	50 (12.5)	105, *p* = 0.275	− 1.16	0.20

*BAI* Beck Anxiety Inventory, *BDI-ii* Beck Depression Inventory-Version ii, *WSAS* Work and Social Adjustment Scale. *10MWT* 10-Meter Walk Test, *TUG* Timed Up and Go, *BBS* Berg Balance Scale

^*1*^*FU, follow-up*

^*2*^*Denotes statistical significance following Mann–Whitney U tests*

Comparisons of outcome measure scores taken at week 1, week 8, and 3-month follow-up are shown in Table 6. Patient-rated measure results indicated statistically significant improvements over time on the BAI, BDI-II, WSAS (Supplemental Fig. 1).

**Table 6:** Median scores and non-parametric analysis of variance across time-points (week 1, week 8, and 3-month follow-up)

	*N*	Week 1	Week 8	3-Month follow-up	Statistical Significance	Effect size
					Friedman's ANOVA	Kendall’s W
		(1)	(2)	(3)	(*χ*^2^, *p* value)	
BAI	31	30 (16)	19 (20.5)	24 (18.5)	15.35, *p* < 0.001	0.248
BDI-II	31	34 (15)	19 (26.5)	23 (27)	24.61, *p* < 0.001	0.397
WSAS	31	29 (9)	22 (16.5)	24 (15)	14.64, *p* < 0.01	0.236
10MWT (m/s)	21	0.81 (0.38)	1.13 (0.45)	1.10 (0.39)	14.80, *p* < 0.01	0.352
TUG (s)	21	13.30 (10.9)	8.21 (6.15)	9.33 (5.61)	17.24, *p* < 0.001	0.410
BBS	21	51 (26)	56 (11)	55 (9)	2.92, *p* > 0.05	0.070

*BAI* Beck Anxiety Inventory, *BDI-ii* Beck Depression Inventory-Version ii, *WSAS* Work and Social Adjustment Scale, *10MWT* 10-Meter Walk Test, *TUG* Timed Up and Go, *BBS* Berg Balance Scale

Clinician-rated physiotherapy outcome measures also showed statistically significant improvements on the 10MWT and TUG, although there were no significant changes in performance on the BBS (Supplemental Fig. 1). Descriptive statistics indicated that of the patients who attended the 3-month follow-up, 45% of patients could mobilize unaided prior to program attendance, with 84% able to mobilize unaided at follow-up.

Post hoc Bonferroni-corrected Wilcoxon signed-rank tests (Table 7) indicated that improvements were significant between week 1 and week 8 for the BAI, BDI-II, WSAS, 10MWT, and TUG. Significant improvements were also present between week 1 and 3-month follow-up for these measures. The magnitude of corresponding effect sizes for significant improvements ranged from ‘moderate’ to ‘large’ [49].

**Table 7:** Post hoc Wilcoxon signed-rank tests comparing time-points

	Week 1 compared to week 8			Week 1 compared to 3-month follow-up			Week 8 compared to 3-month follow-up		
	*Z*	*P*^*1*^	Effect siz*e*	*Z*	*P*^*1*^	Effect size	*Z*	*P*^*1*^	Effect size
BAI	− 3.54	< 0.001	− 0.45	− 3.17	< 0.01	− 0.40	− 0.14	> 0.05	− 0.02
BDI-II	− 3.94	< 0.001	− 0.50	− 3.57	< 0.001	− 0.45	− 1.25	> 0.05	− 0.16
WSAS	− 3.61	< 0.001	− 0.46	− 2.97	< 0.01	− 0.38	− 0.61	> 0.05	− 0.08
10MWT (m/s)	3.02	< 0.01	0.47	2.75	< 0.01	0.42	0.02	> 0.05	0.00
TUG (s)	− 3.08	< 0.01	− 0.47	− 3.41	< 0.01	− 0.53	0.89	> 0.05	0.14
BBS	1.51	> 0.05	0.23	1.10	> 0.05	0.17	0.77	> 0.05	0.12

*BAI* Beck Anxiety Inventory, *BDI-ii* Beck Depression Inventory-Version ii, *WSAS* Work and Social Adjustment Scale, *10MWT* 10-Meter Walk Test, *TUG* Timed Up and Go, *BBS* Berg Balance Scale

^1^Denotes statistical significance following Bonferroni correction for multiple comparisons

## Discussion

The FiND program aimed to deliver a specialist multidisciplinary rehabilitative program in an outpatient setting and offer an alternative treatment pathway to inpatient admission for FND. The program sought to reduce symptom severity and improve the functional mobility of attending patients. Both patient- and clinician-rated/performance measures were selected to assess the effectiveness of the program as delivered in routine clinical practice, in accordance with expert recommendations [7, 38].

### Outcomes and effectiveness

Significant improvements in scores were present when comparing week 1 to week 8 for the BAI, BDI-II, WSAS, 10MWT, TUG and BBS, and when comparing week 1 to 3-month follow-up for all measures aside from the BBS. Comparisons between week 8 and 3-month follow-up did not indicate significant changes in these outcome domains, suggesting that improvements were maintained during this period. Effect sizes ranged from moderate to large, suggesting that these improvements were likely to be clinically significant, supporting the effectiveness of the FiND program.

Comparisons between baseline patient-rated measures and those collected at 3-month follow-up show that at the beginning of the program, most patients were classified as having severe anxiety, depression, and functional impairment; whereas at 3-month follow-up, the percentages of patients scoring in the severe categories had reduced significantly. These findings may have important implications, as factors such as mood, anxiety, and illness perceptions are closely linked to quality of life in FND [30].

These results accord with outcomes from alternative MDT rehabilitative programs for FND, which also reported improvements in mood, anxiety, and functional ability following attendance [27, 32, 35]. The magnitude of effect sizes reported from such programs also suggests clinical significance, with effect sizes that ranged from small to moderate for a 5-week outpatient intervention [35], and those that ranged from small to large for a 4-week inpatient intervention [27]. Symptom duration at baseline also compared with other outcome studies, with mean symptom duration of 6.5 years for a 5-week intervention [35] and mean symptom duration of 4.8 years for a 4-week inpatient intervention [27].

Together, the existing findings indicate the potential clinical utility of MDT interventions for FND and provide further support for the provision of such interventions in outpatient settings. There are several features of our program which may have contributed to the favorable outcomes and large effect sizes observed. First, the program is relatively intensive and extended in duration, relative to others described in previous reports [27, 32, 35]. The unique combination of individual and group-based sessions, psychotherapy, and physiotherapy, as well as an emphasis on self-management of symptoms, may also represent particular strengths of this program. The involvement of former service-users and friends/family of current service-users may also have contributed to the beneficial effects of this intervention.

Despite the positive findings reported, a proportion of patients still scored in ‘severe’ categories on outcome measures at 3-month follow-up, indicating that such individuals could benefit from further intervention. As such, it is common for subsequent recommendations or referrals to be made to appropriate services following FiND program attendance, in the hopes that improvements made can be consolidated and extended. Similarly, improvements demonstrated throughout the program may better equip patients for subsequent input from additional services, as some individuals may have initially been declined from such services due to their level of impairment.

### Comorbidities

A large proportion of patients (78%) had one or more psychiatric comorbidity, including diagnoses of depression, obsessive-compulsive, post-traumatic stress, generalized anxiety, and/or personality disorders. This is consistent with previous findings and suggests that our sample was representative of the broader FND population [8, 50–55]. Given the small number of patients without psychiatric comorbidities in our cohorts, it was not possible to conduct sensitivity analyses to assess the possible influence of such diagnoses on outcomes.

Pain and fatigue were amongst the most common symptoms reported at baseline, indicating that these symptoms are important to address through the MDT approach. The presence of comorbid chronic pain has been inversely correlated with clinical outcomes in FND [56].

A large proportion of patients were taking one or more pharmacological treatment (i.e., including psychotropic and pain medications), which could influence treatment effectiveness. For example, a randomized clinical trial for functional seizures demonstrated greater improvement in outcomes (quality of life, depression, anxiety, somatic symptoms, psychosocial functioning) in a CBT-only arm compared to a CBT plus sertraline arm, which could be due to medication adverse effects [24]. Whilst we were able to retrieve data on pharmacological treatments at baseline, this information was not available for the subsequent time-points; therefore, it is possible that changes in medication status may have influenced the outcomes in our cohorts.

### Limitations

Despite the severity of symptom presentation in patients who attended the program, there may have been some degree of sampling bias in the patients included in these analyses. Our extensive eligibility criteria and the outpatient nature of the program could mean that our cohorts were generally less complex in their presentation and had better prognosis, with the suitability assessment in part examining the patients’ readiness and willingness to undergo intensive rehabilitation. Furthermore, we were able to include only a modest sample size. We, therefore, cannot conclude that these outcomes would be generalizable to the broader FND population and additional studies in larger, more diverse samples are needed.

The lack of FND symptom-specific outcome measures is also a limitation of this evaluation. Notably, there is currently no single outcome measure suitable for use across all FND symptom types, and no widely endorsed symptom-specific tool for any FND subtype, thereby limiting the selection of outcome measures [38]. We also did not collect clinician or patient-rated outcome scores using the Clinical Global Impression-Improvement Scale (CGI-I) which has been shown to be sensitive to change in FND populations [38].

Our 3-month follow-up period is relatively short compared to other published reports, making it difficult to draw conclusions about long-term treatment outcomes in our cohorts. Nevertheless, both 4- and 5-week MDT-based outpatient programs previously found that improvements were largely sustained at 6- and 12-months follow-ups [27, 35].

Our program did not include input from occupational therapy, which is a potentially important omission that may have limited the effectiveness of the intervention, particularly regarding outcomes relating to functional impairment.

Although we report findings from physiotherapy outcome measures, we acknowledge that changes in mobility-aid requirement throughout the course of the program could have impacted the validity of such measures. In some cases, baseline measures were collected while a walking-aid (e.g., walking stick, crutches) was in use, whereas subsequent task performance may have been assessed unaided, provided that individual improvement in mobility allowed this. For this reason, physiotherapy outcome measures may not adequately demonstrate improvements gained, as they refer to walking speed and balance, which is likely to have been affected by the presence or absence of a walking-aid. As input from physiotherapy was an integral part of the MDT program provided, it was deemed appropriate to include these measures in the analysis, although subsequent evaluation of the program would benefit from a standardized approach when using these measures, as well as adoption of a valid and reliable measure of mobility aid to include in the analysis.

The implications of these findings are limited as they were derived from retrospective, routinely collected data, rather than a prospective, randomized controlled research study. We were unable to take precautions to control for common sources of error, as would be afforded by more stringent, prospective research methods. There was no use of a comparison group/intervention or placebo which could allow better assessment of the relationship between the intervention and improved outcomes. As such, it is possible that extraneous factors may have been influential, and thus we cannot infer with certainty that the outcomes achieved were wholly attributable to the intervention provided.

### Future directions

It would be beneficial to conduct further studies into the efficacy of outpatient MDT treatment for FND in controlled trials, with our initial findings serving as a positive indication of prospective findings. Further studies with different designs are also warranted to assess which components of the program led to which gains. Despite being unable to discount the influence of extraneous factors and possible selection biases, these initial findings offer ecological validity as they reflect outcomes of an intervention provided in a naturalistic setting, offering insights into potential real-world benefits to patients. Inclusion of an additional follow-up session, i.e., at 6 or 12 months, would allow us to assess whether improvements were sustained in the long-term.

## Conclusions

Our findings suggest that this outpatient MDT program provided benefit to patients regarding overall reduction in physical and psychological symptom severity, improvement in functional mobility, perceptions of functional impairment, and performance on clinician-rated physiotherapy outcome measures. Importantly, these findings provide additional support for the clinical value of a multidisciplinary outpatient approach to treating FND and suggest that further investigation with controlled studies is needed.

### Supplementary Information

Below is the link to the electronic supplementary material.

Supplementary file1 (DOCX 399 KB)

## Data Availability

The data will be made available on reasonable request.
