# Supplementary material for: Routine outcomes and evaluation of an 8-week outpatient multidisciplinary rehabilitative therapy program for functional neurological disorder
Source: J Neurol. 2023 Dec 13;271(4):1873–84. doi: 10.1007/s00415-023-12111-4 (PMC10973040; doi:10.1007/s00415-023-12111-4)
Supplement: Supplementary file 1 — Supplementary file1 (DOCX 399 KB) [file 415_2023_12111_MOESM1_ESM.docx]

**
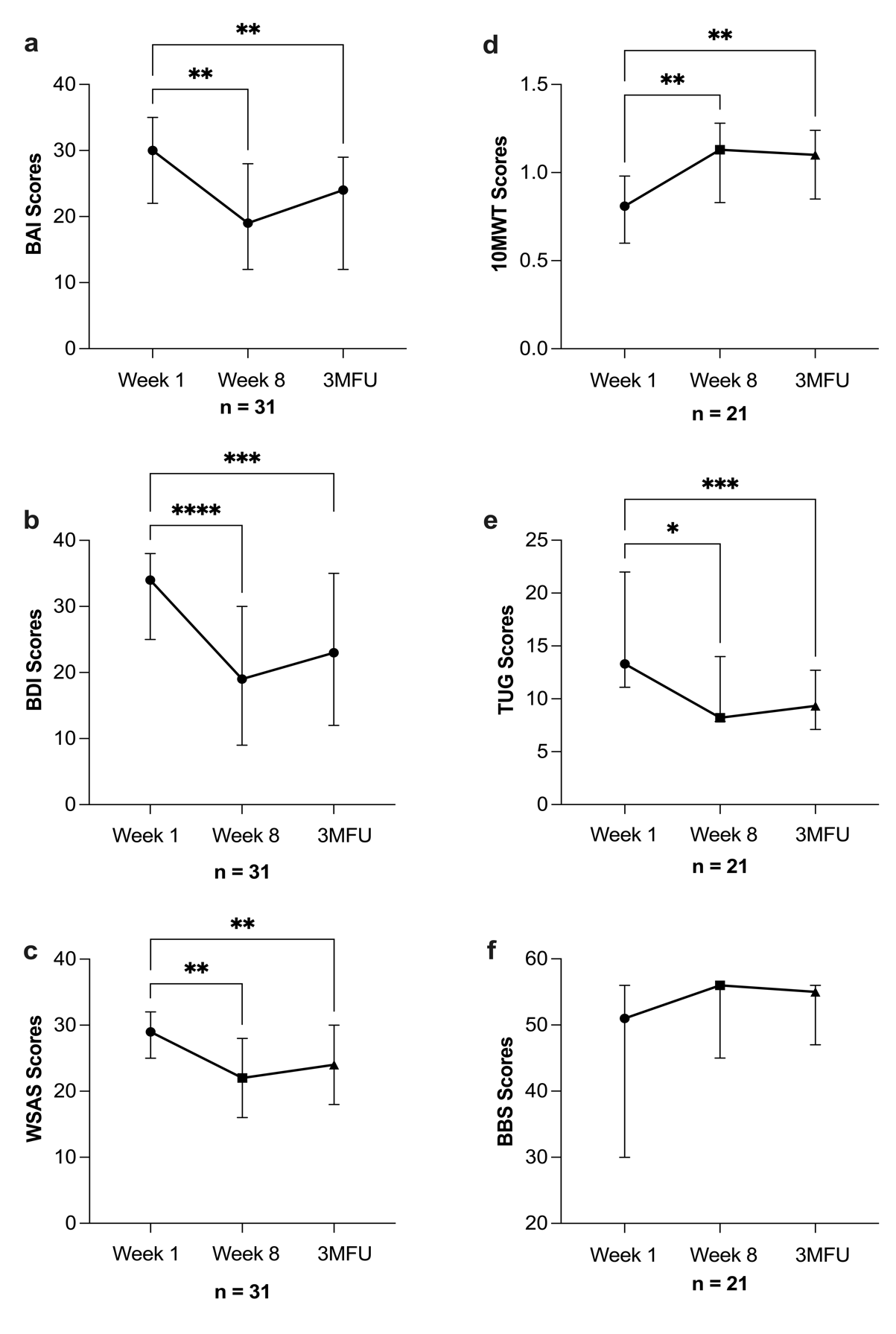
**

**Supplemental Figure 1.** Patient and clinician-rated outcomes at week 1, 8, and three-month follow-up (3MFU) showing changes in **a** median BAI scores **b** median BDI scores **c** median WSAS scores **d** median 10MWT scores and **e** median TUG scores. **f** shows no changes in median BBS scores. Error bars denote the 95% confidence interval.
